# Mechanical and Solvothermal Recycling of End-of-Life Carbon Fibre-Reinforced Plastic Products: Process Feasibility and Flexural Performance of Recycled Composites

**DOI:** 10.3390/polym17070878

**Published:** 2025-03-25

**Authors:** Dario De Fazio, Luca Boccarusso, Antonio Formisano, Rossella Grappa, Giuseppina Luciani, Francesco Branda, Massimo Durante

**Affiliations:** Department of Chemical, Materials and Production Engineering, University of Naples “Federico II”, P.le Tecchio 80, 80125 Naples, Italy; luca.boccarusso@unina.it (L.B.); aformisa@unina.it (A.F.); rossella.grappa@unina.it (R.G.); luciani@unina.it (G.L.); branda@unina.it (F.B.); mdurante@unina.it (M.D.)

**Keywords:** carbon fibre-reinforced plastics, composite recycling, mechanical recycling, chemical recycling, FTIR analysis, mechanical properties

## Abstract

The increasing demand for high-performance materials has led to an increase in the use of carbon fibre-reinforced plastics (CFRPs) in recent decades, increasing the waste from end-of-life materials and off-cuts. The recycling of CFRPs, especially when thermosetting matrices are used, still remains an open challenge for academia and industry, with chemical, thermal and mechanical strategies being explored. Among them, mechanical methods have garnered growing interest since they do not require high specific energy consumption or expensive apparatus. However, from the literature it was observed that when using these methods, traces of old matrix remain on the fibre’s surface, compromising the fibre–matrix adhesion efficiency and limiting their use in recycled composites. On the other hand, solvothermal methods are known for their high matrix dissolution efficiency that in turn improves the fibre–matrix adhesion. Therefore, in this paper, end-of-life CFRPs from the aeronautic sector were machined using a milling-based mechanical recycling method, while to remove the residual matrix from the fibre surface, the recovered chips were chemically treated with a two-step treatment at low temperature. Then, two types of recycled composite laminates were manufactured using the compression moulding technique: the first using recycled fibres only from the mechanical recycled method, and the second one using recycled fibres deriving from both recycling methods. The feasibility of the process was analysed observing that the additional chemical treatment led to a mass loss of almost 24% in the recycled fibres. FTIR analysis revealed the complete matrix dissolution since no spectra of epoxy resin groups were detected. Finally, the flexural behaviour of the recycled composites was investigated, revealing an increase in the flexural strength and modulus of the second sample typology, respectively, of almost 42% and 76% thanks to the improved fibre–matrix adhesion as a consequence of the solvothermal treatment.

## 1. Introduction

Composite materials have attracted a growing interest in the industrial field thanks to their properties in terms of excellent mechanical performance, light weight and high design of freedom. Among them, carbon fibre-reinforced plastics (CFRPs) have attracted the greatest interest for their superior mechanical performance and low weight. These characteristics promoted their use in widespread application fields like construction, sports, energy, automotive, naval, aeronautic and so on [1,2,3,4,5,6,7]. On these premises, the demand for CFRPs has grown significantly. Indeed, between 2010 and 2020, their demand was tripled, reaching approximately 170 kTons [7,8,9,10,11,12,13]. Some projections indicate that this value inexorably increases up to 190 kTons by 2050 [7,8,9,10,11,12,13].

Then, the increasing use of composite materials in the industrial world will inexorably increase the amount of waste that derives from their use. Usually, the production phases of composite materials and end-of-life products are mainly considered as principally responsible for waste formation [8,14,15,16]. Particular attention must be paid to end-of-life components since composite materials, thanks to their mechanical and physical characteristics, revealed a long-term service life [17,18,19,20]. Therefore, this aspect leads to the observation that all the composite components that entered into service some decades ago, are close to be decommissioned and dismissed. However, despite this, landfilling remains the most common and cost-effective disposal method. Therefore, it is possible to conclude that there is a strong need for defining sustainable and appropriate recycling strategies to mitigate their environmental impact [21,22,23,24].

Based on these considerations, at the actual state, the life cycle of composite materials follows a linear model, starting from production and ending with disposal. To address this issue, European directives 2008/98/EC and 2000/53/EC set clear recycling targets, requiring at least 70% of waste materials and 85% of end-of-life vehicles to be recycled. These regulations aim to reduce landfill dependency and promote a circular economy [25].

Then, both the industrial and academic worlds are moving toward the recycling of composite materials, observing that this process strongly depends on the nature of the matrix used. Interest has been shown in the recycling of composites with a thermoplastic matrix [26,27,28,29,30]. However, the recycling of thermosetting composites is an open challenge due to the presence of cross-links within the matrix molecule and, in case of the aeronautic composite materials analysed in this paper, the presence of flame retardant agents.

To recycle waste composite materials, three main recycling strategies have been developed. They can be classified into thermal, chemical and mechanical methods. With the thermal recycling process, composite materials are subjected to high temperatures to degrade the matrix and recover the fibres. This process includes pyrolysis which uses high temperatures and fluidised bed that uses a combination of high temperature and fluids to allow for the matrix decomposition into solid, gasses and char [31,32,33,34,35,36,37]. The chemical recycling method, known as solvolysis, dissolves the thermoset resin into the basic monomers using chemical solutions in sub-critical or super-critical conditions. The process efficiency is ensured by the working parameters in terms of pressure and temperature. These conditions allow for a good penetration of the solution within the composite material ensuring a good matrix dissolution [38,39,40,41,42,43]. Despite the thermal and chemical recycling strategies, the mechanical method allows for composite recycling without the use of high temperatures or chemical agents. However, even though the mechanical recycling method presents some advantages since heat and chemical agents are not required, it also shows some limitations in terms of recycled fibre length. With shredding, hammer milling, milling and grinding processes are usually obtained short fibres in a range between 50 µm and 30 mm. The chips are sieved to allow for a separation based on their size. However, literature confirms that the mechanically recycled fibres present traces of old matrix on their surface [44,45,46,47,48]. This aspect, when used as reinforcement in recycled composite materials, allows for a reduced adhesion at the fibre–matrix interface. The reduced adhesion efficiency, together with the reduced fibre length, contributes to compromising the overall mechanical properties of the recycled composites, limiting their application as load-bearing parts.

Therefore, based on these considerations, it could be of interest to combine multiple recycling methods to hybridise the recycling process and improve some drawbacks, such as the reduced adhesion efficiency at the fibre–matrix interface and the short fibre length typical of the mechanical recycling method. To this end, it is known from the literature that both the thermal and chemical recycling processes are characterised by a high level of recycling efficiency, which allows for clean recovered fibres [49,50,51,52]. However, these methods require a large amount of energy since they demand high temperatures to recycle composite materials [49,50,51,52]. In addition, even though numerous research activities are based on the recycling of thermosetting matrix composite materials, there is increasing interest in the use of flame-retardant phases within the thermosetting resins in the composite system. Many activities are focused on this topic. For example, Huo et al. [53] examined the state-of-the-art use of phosphorus as a flame-retardant agent within a thermoset epoxy resin, analysing its effect on the flame retardancy, thermal stability and mechanical properties of the resulting resin. However, some challenges emerged from its use that require additional investigations. These challenges are related to its potential impact on human health in terms of toxicity and the feasibility of a recycling strategy aiming to use a more sustainable material. Velencoso et al. [54] and Liu et al. [55], as well as Huo et al. [53], investigated the use of polymeric materials with added phosphorus, observing that this material is particularly effective in both gas and condensed phases in the case of fire. It was found that phosphorus is highly versatile, can be considered a potential substitute for halogenated variants, and it has the potential to fulfil the criteria for future flame retardants in both bio-based and recyclable materials.

However, multiple flame-retardant agents can sometimes be combined to achieve a synergistic effect on flame resistance. Zheng et al. [56] analysed the state-of-the-art use of phosphorus and ammonium polyphosphate as flame-retardants. They observed that their combination resulted in improved hydrophobic properties and reduced smoke production in case of fire, while preserving the mechanical properties of the composite material. Yang et al. [57] studied the combination of expandable graphite and phosphorus as flame-retardant agents. During the experimental campaign, research activities such as thermogravimetric analysis (TGA), vertical burning, cone calorimeter and Fourier-Transform Infrared (FTIR) analysis were performed on the composite material. From the tests, the authors confirmed the presence of graphite structures and carbonised resin characterised by strong interfacial bonding that enhances the barrier effect in the condensed phase.

Wu Klinger et al. [58] investigated the state-of-the-art in recycling thermoset matrix with flame-retardant agents. In detail, the study was focused on polymers characterised by specific bonds within the structure that can be broken and reconnected reversibly when subjected to external stimuli such as the thermomechanical recycling method. This characteristic makes the thermomechanical process an interesting method for recycling complex materials. However, at the actual state, further investigations are required since the authors pointed out that high concentrations of these adaptable bonds can compromise the thermal stability and the mechanical properties of the final material.

A literature overview reveals that the use of flame-retardant agents within polymeric resins is a current and relevant topic. In some studies, the necessity to adopt appropriate recycling strategies emerged to promote more sustainable materials. In this context, although characterised by high recycling efficiency, thermal methods like pyrolysis are not suitable and efficient for these materials. On the other hand, the chemical recycling method can be considered an interesting strategy since it allows for the recovery of both the fibrous reinforcement and the polymer matrix [59]. However, despite the increasing interest in the use of flame-retardant agents within thermoset matrices, there are still limited activities focused on the chemical recycling processes of flame-retardant-added polymeric materials.

The literature overview further highlights that chemical recycling processes usually require high temperatures to generate high pressure within the reactor, promoting the swelling of the recycling composite material and improving the solution’s penetration between the layers to achieve better dissolution of the old matrix. It is noteworthy that in this research work, the chemical recycling method is used after the mechanical one on recovered material in the form of fibre bundles. Therefore, the reduced dimension of the recycled material promotes the swelling of the recovered material and the penetration of the chemical solution within the fibre bundles under soft treatment conditions.

Based on these observations, this study explores the addition of a further chemical treatment on mechanically recycled CFRP materials, aiming to improve the adhesion efficiency at the fibre–matrix interface. The chemical treatment is divided into two steps: the first one is based on an acetic acid solution and the second one on an acetone solution. Both chemical treatments were performed at 150 °C, far from the typical temperatures adopted during solvolysis, aiming to use the same temperatures reached during the production of composite materials in an autoclave. At the end of the recycling processes, FTIR analyses were performed on both the solvothermally treated and untreated chips to assess the efficiency of the process. Then, the mechanical properties in terms of flexural performance of the recycled composite materials reinforced both with the treated and untreated fibres were investigated through three-point bending tests.

## 2. Materials and Methods

### 2.1. Materials and Sample Preparation

Since the aim of this research activity is focused on the combination of the mechanical recycling method with a solvothermal recycling strategy performed at a temperature typical of composite production in an autoclave, it was decided to use a carbon fibre prepreg that derives from the industrial sector. In detail, the CFRP material derives from the aeronautical field, which imposes stringent regulations in terms of material fire resistance. Therefore, during the prepreg production, fire-retardant polymeric materials were added to the epoxy matrix, which in turn makes the chemical recycling process more complex. Based on these considerations, this material can be considered a typical end-of-life product that derives from the industrial field. Therefore, to simulate the recycling process of an aeronautical component at the end of its service life, the CFRP prepreg was used for the production of a composite laminate and then it was recycled. The CFRP material was produced using a 200 g/m^2^ twill weave carbon/epoxy prepreg supplied by Geven S.p.a. (Nola, Italy). Each layer is characterised by an overall thickness of almost 0.2 mm; therefore, a total of 20 layers were stacked in a [0/90]s configuration to produce laminates with dimensions 200 × 200 × 4 mm^3^. They were manufactured using a compression moulding technique. Each layer was placed on a plate mould, and at the end of the stratification phase, the composite was cured at 150 °C under a pressure of almost 0.8 MPa for 8 h.

The produced CFRP laminates were characterised by a density of 1.5 g/cm^3^, a fibre volume content in a range of 60–70% and a flexural strength and elastic modulus, respectively, of almost 700 MPa and 50 GPa. Among all the mechanical properties, the flexural ones were taken into account since these mechanical performances are the main aim of the present research work.

The recycled composite samples were manufactured adopting the same production strategy used for the fabrication of the CFRP laminates. To do that, layers of carbon fibre chips were stacked and impregnated with resin films. However, the recycled fibres were impregnated using a flame-retardant added Letoxit LFX 56 epoxy resin supplied by Mates s.r.l. (Cisliano, Italy).

### 2.2. Recycling Method

**Figure 1:**
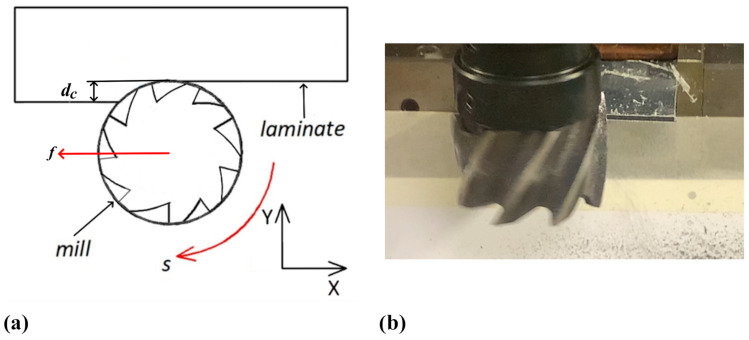
Mechanical recycling operation: schematisation (**a**) and execution of a test (**b**).

CFRP laminates were then subjected to a mechanical recycling method based on the milling process to produce recovered carbon fibre chips. To do this, a 5-axis CNC machining centre (C. B. Ferrari) equipped with an HSS end mill tool 40 mm in diameter was used to perform the peripheral milling process. In Figure 1, a schematisation (Figure 1a) and the execution of a mechanical recycling operation (Figure 1b) are reported. A well-defined set of cutting parameters was adopted during the milling process to allow for a high material removal rate (MRR). A spindle speed (*s*) of 200 rpm, a feed rate (*f*) of 1000 mm/min and a depth of cut (*dc*) of almost 5 mm were chosen for the mechanical recycling process [60,61]. The use of the defined set of cutting parameters derives from research activities focused on the effect of their combination on the recovered chip size, as well as on the cutting power required during the machining operations and the cutting energy demand. It was observed that the adoption of low *s* values in combination with high *f* and *dc* promotes high *MRR* values and the formation of larger chips, while also achieving the lowest specific cutting energy values [60,61].

**Figure 2:**
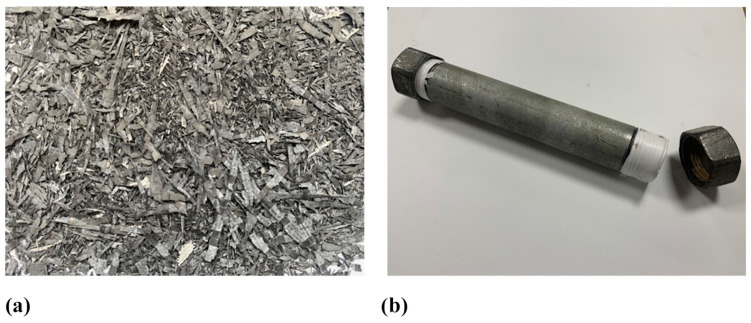
Sieved recycled carbon fibres before the chemical treatment (**a**) and the chemical reactor vessel (**b**).

At the conclusion of the machining process, the recovered material was sieved using a shaking table to separate the chips based on their size. The material was then classified into three categories: powders with a length of less than 0.2 mm, fine fibres ranging from 0.2 to 20 mm and coarse fibres exceeding 20 mm in length. Based on previous authors’ research activities [60,61,62], only fine fibres were selected for the production of new recycled composite materials (Figure 2a). To study the effects of chemical treatment, only a portion of these fibres was subjected to a chemical process consisting of two steps. The chemical treatment is described below:

- In the first step, the recovered fibres were treated with an 11% acetic acid solution by volume to promote the swelling of the chip material. The fibres were placed in a Teflon-lined stainless-steel reactor vessel measuring 200 mm in length and 28 mm in diameter (Figure 2b). The vessel was sealed with the chemical solution, maintaining a fibre-to-solution weight ratio of 1:5, and heated in an oven at 150 °C for 8 h. Then, the reactor was removed and allowed to cool down naturally. Once the chemical treatment was completed, the recovered fibres were thoroughly washed with acetone.
- In the second step, the reactor vessel was sealed with a pure acetone solution, maintaining the same fibre-to-solution weight ratio of 1:5. As in the first step, the treated fibres were sealed in the reactor and heated in an oven at 150 °C for 8 h. Following this treatment, the vessel was allowed to cool naturally, and the fibres were washed again with acetone to remove any residual traces of dissolved matrix from their surface.

The use of a low temperature solvothermal recycling process can be associated with the possibility of using the same temperatures reached during a typical autoclave production technique. This, as well as the use of easily available chemical solutions, makes the solvothermal process and the mechanical one easily implementable in the industrial sector.

**Figure 3:**
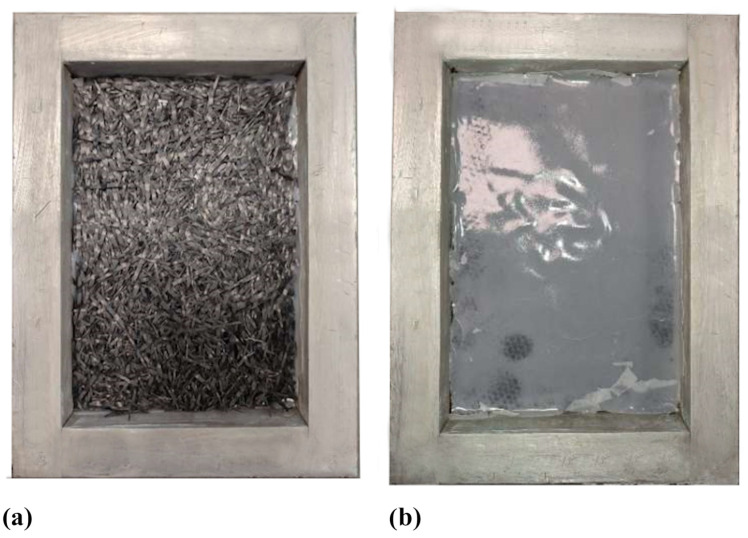
Detail of the production phase where the recovered fibre layers (**a**) are impregnated with the epoxy resin film (**b**).

Both the treated and untreated recovered fibres were used to produce new composite materials (respectively labelled as T-RC and U-RC) by means of the compression moulding technique. Figure 3 shows some of the production phases where both the treated and untreated recycled fibres were stacked (Figure 3a) and impregnated (Figure 3b) with the flame-retardant added resin films. Its choice is related to the fact that the category of recycled composite materials produced following the recycling processes can be reused and reintroduced in industrial sectors as secondary components since they are reinforced with short fibres. Therefore, since this study is aimed at the recyclability of end-of-life composite materials from the aeronautic sector, it was decided to use a resin film with added flame-retardant phases as the new matrix.

During the production phases, layers of resin film alternated with layers of recycled chips were stacked in a 150 × 100 mm^2^ rectangular mould and placed in a hydraulic press to allow for the curing of the recycled composites at 130 °C under a pressure of 0.3 MPa for 2 h. By adopting this production strategy, for both sample types, the reinforcement fraction was kept constant, fixing this value to approximately 35% by weight, considering the overall fibre–matrix composition. Table 1 presents the main geometrical and physical details of the two recycled composite samples used in this process.

### 2.3. Chemical Characterisation

To evaluate the efficiency of the two-step soft chemical treatment in terms of matrix removal from the surface of the fibrous reinforcement, two main parameters, the swelling ratio (*SW*%) and the mass loss (*ML*%), were used. To do this, the weight of the recycled material was measured before and after the chemical treatment, and then the two parameters were evaluated, respectively, through the following equations:

(1) $${SW}_{\%}=\left(\frac{M_{w}-M_{d}}{M_{b}}\right)\times 100$$

(2) $${ML}_{\%}=\left(\frac{M_{b}-M_{d}}{M_{b}}\right)\times 100$$

where *M_b_* corresponds to the mass of the recycled fibres before the chemical treatment, *M_w_* is the wet mass that includes the same amount of recycled material and the mass of the chemical solution, and *M_d_* is the mass of the dried treated recycled material at the end of the chemical treatment.

A further spectroscopic analysis was performed on the surface of the chemically treated fibres, which in turn allows for the identification of spectra indicative of the presence of traces of the old matrix. For this purpose, a FTIR spectroscopy in the attenuated total reflection (ATR) analysis was performed to analyse the chemical composition of the T-RC samples using a Nexus FTIR Spectrometer. The ATR absorption spectra were collected using a DuraSam-plIR II accessory equipped with a ZnSe Cristal in the range of 4000–400 cm^−1^ with a spectral resolution of 8 cm^−1^.

### 2.4. Mechanical Characterisation

The mechanical properties of both sample types were investigated by three-point bending tests performed in accordance with the ASTM D790 standard using a universal testing machine, Instron 5565, equipped with a 5 kN piezoelectric load cell. A total of three valid specimens for each sample were tested by fixing the span-to-thickness ratio at 32.

Stress-strain flexural curves were determined by evaluating the flexural stress (*σ_f_*) and the flexural strain in the outer surface (*ε_f_*) by the following equations:

(3) $$\sigma_{f}=\frac{3Pl}{2bt^{2}}$$

(4) $$\varepsilon_{f}=\frac{6Dt}{l^{2}}$$

where *P* is the load, *l* is the support span, *b* and *t* are the width and thickness of the specimens and *D* is the deflection of the centre of the specimens.

From the bending tests, the flexural strength (*σ_f_*_,*max*_, i.e., the maximum flexural stress) and the flexural modulus (*E_f_*, using the chord method described by Equation (5)) were evaluated:

(5) $$E_{f}=\frac{\left(\sigma_{f2}-\sigma_{f1}\right)}{\left(\varepsilon_{f2}-\varepsilon_{f1}\right)}$$

where *ε_f_*_1_ and *ε_f_*_2_ are the flexural strains measured at defined points of the flexural curve and *σ_f_*_1_ and *σ_f_*_2_ are the corresponding flexural stresses.

## 3. Results

### 3.1. Mechanical Recycling

The milling process parameters adopted in this experiment promoted the formation of fine and coarse fibres over powders. This outcome is a direct consequence of the specific machining conditions, which promote a high chip thickness per tooth, favouring the generation of fine fibres instead of powders and coarse fibres, thereby reducing the proportion of the shortest chip types. The data revealed a substantial formation of fine fibres, which amounted to approximately 75.10% of the overall recovered material. On the other hand, powders and coarse fibres were, respectively, around 16.20% and 8.70% of the total machined material. This fibre distribution, with the prevalence of fine fibres, can be attributed to the adopted machining parameters, and more specifically to the low *s* compared to *f*. When combined with the adopted *f* and *dc*, these parameters lead to an increase in the removed chip thickness, favouring the production of fine fibres. These process conditions minimise the laminate fragmentation into powders and coarse fibres, customising the recycling process.

### 3.2. Chemical Results

At the end of the solvothermal chemical treatments, it can be pointed out that the adoption of the defined process parameters, in conjunction with the reduced size of the recycled fibres, promotes the penetration of the chemical solution within the fibre bundles, resulting in a high *SW*% value (55.20%) and consequently in a high interaction between the old matrix and the chemical solution. This aspect promotes the matrix dissolution from the surface of the recycled fibres, leading to an overall weight reduction of the treated material. Therefore, based on these considerations, at the end of the chemical process, a reduction in the chip’s mass was observed, resulting in a *ML*% value of approximately 24%. This value is justified by the prepreg used during the experimental campaign to produce the machined laminates, which is characterised by a fibre’s volume fraction in the range of 60–65%. Therefore, although the chemical process allows for high matrix removal, it is not possible to achieve higher values of *ML*%.

**Figure 4:**
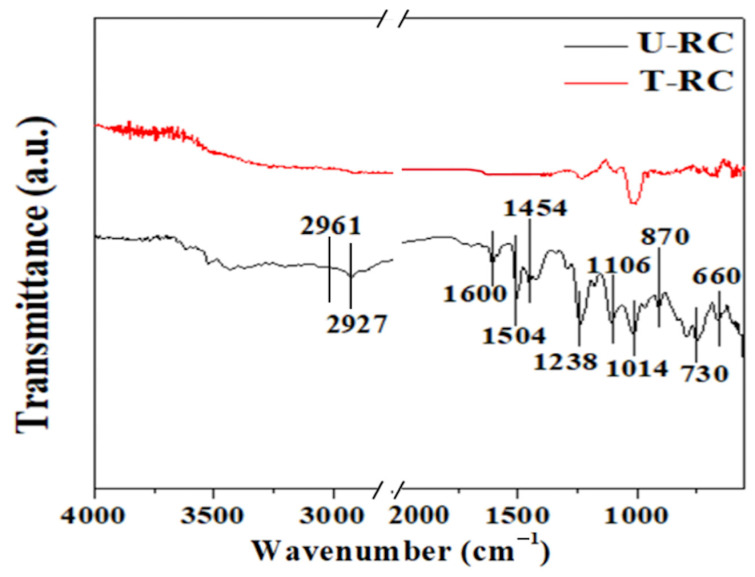
FTIR spectra of U-RC and T-RC samples.

What was pointed out about the high efficiency of the two-step soft chemical recycling method is further corroborated by the results observed from the FTIR analysis. In detail, Figure 4 reports the spectra of both samples under inspection.

In this figure, it is possible to observe, especially in the U-RC case, the presence of bands localised at different wavenumbers corresponding to the presence of some functional groups on the fibre’s surface. Focusing on the U-RC sample, some bands at 1600, 1504, 1454, 1238 and 1014 cm^−1^ appear in its spectrum, revealing the nature of the matrix since they are typical of all epoxy resins [59,63]. In more detail, the first three peaks may be attributed to the benzene ring, and the band at 1238 cm^−1^ is ascribed to the aryl alkyl ethers (C-O-C) group. Meanwhile, the band at 1014 cm^−1^ may be attributed to the presence of C-OH groups formed during the cure. The bands at 2961 cm^−1^ and 2927 cm^−1^ are due to -CH3 and -CH2- groups [63]. The band above 3000 cm^−1^ is due to stretching vibration of the OH group. The wavenumber range 2000–2800 cm^−1^, where no bands were detected, was cut off. The other bands visible in the spectrum of the untreated sample might be attributed to atomic groups that characterise the constituents used in the flame-retardant phases. This observation is further supported by the fact that these additives are usually introduced into the polymeric matrix system to satisfy the stringent fire resistance regulations required for the application of these materials in sectors like the aeronautic field.

On the other hand, looking at the spectrum of the T-RC sample, it is possible to observe that it dramatically changes because of the solvothermal recycling process. The typical bands that identify the epoxy matrix completely disappear, meaning that the old matrix is removed from the surface of the recovered fibres. However, the band at 1014 cm^−1^, together with the one above 3000 cm^−1^, typical of the C-OH group, is still visible, indicating that partial oxidation on the fibre’s surface occurs [64].

Therefore, from the analysis of the spectra of both samples, it is possible to assert that the epoxy structure characterising the used matrix has been destroyed by the solvothermal process. A large part of the bands typical of the flame retardants is not visible on the spectrum of the treated material because of their removal, and the absence of the peaks typical of the epoxy resin reveals that the matrix is dissolved.

The traces of oxygen that derive from the flame-retardant agents and from the partial fibre oxidation, as well as the increase in the polar groups found on the surface of the chemically treated fibres, lead to the conclusion that an improvement in the interaction with the functional groups of the new epoxy matrix is expected. This improvement enhances the adhesion efficiency at the fibre–matrix interface, which in turn results in superior mechanical properties of the solvothermal treated chippings obtained through the mechanical process.

### 3.3. Flexural Properties

**Figure 5:**
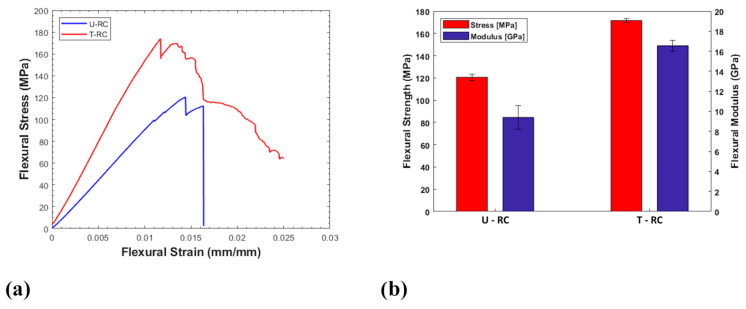
Typical stress vs. strain flexural curves (**a**) and mean values of the maximum flexural strength and modulus (**b**) of each sample family.

The results of the three-point bending tests are reported in Figure 5. In detail, Figure 5a shows the typical stress-strain curves of each family of recycled composites, and Figure 5b shows the mean values of the maximum strength and flexural modulus. From a global overview of the bending curves, it is possible to assert that both families of recycled composites are characterised by a linear stress increase up to the failure of the specimen as a consequence of the applied displacement.

The results from the flexural tests revealed a significant improvement in the mechanical properties of the T-RC sample. This outcome highlights the crucial role of the additional chemical treatment in enhancing the flexural properties of the recycled material. Specifically, the T-RC sample exhibited a notable increase in both flexural strength and modulus, showing improvements of around 42.24% and 76.25%, respectively. Therefore, the increase in mechanical properties can be attributed to the positive impact of the chemical treatment on the fibre–matrix interface, which improved the adhesion efficiency. In contrast, the U-RC sample revealed a failure mechanism dominated by debonding and pull-out, indicative of a weak bonding efficiency between fibres and matrix. These phenomena significantly limit the capacity of untreated fibres to efficiently transfer mechanical stresses, leading to reduced mechanical properties.

Based on these considerations, the soft chemical treatment appears to mitigate these limitations, promoting better surface interaction at the fibre–matrix interface and resulting in improved flexural properties. These assertions are corroborated by the results from the FTIR analysis. In detail, the presence of residual matrix, responsible for the reduced fibre–matrix adhesion in case of the U-RC sample, is clearly highlighted by the presence of the bands between 1600 and 1014 cm^−1^. Therefore, at the end of the additional solvothermal process, the absence of these spectra confirms the complete matrix removal from the fibre’s surface. This improves the fibre’s wettability, promoting an improved adhesion efficiency. This aspect results in the mitigation of the pull-out and debonding phenomena that affected the failure mode of the U-RC sample. These findings highlight the relevance of the chemical modification of the fibre surface in optimising the mechanical performance of recycled composite materials.

**Figure 6:**
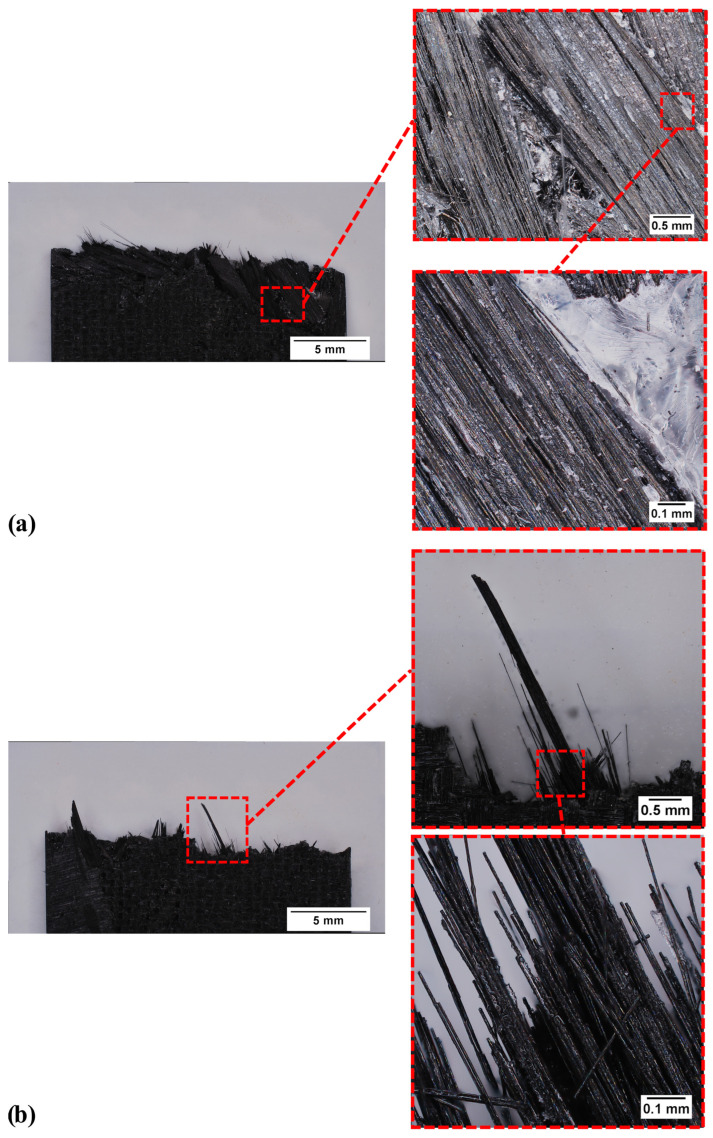
Magnifications of the failure area of flexural specimens from U-RC (**a**) and T-RC samples (**b**).

In Figure 6, the failure regions of both samples are represented. Focusing on the U-RC (Figure 6a), the residues of the old matrix on the fibre surface result in the initiation of pull-out and debonding phenomena near the failure region. This aspect leads to the complete separation of fibre bundles within the sample’s thickness, promoting the formation of new surfaces where the fibre bundles, which do not contain traces of new matrix, are exposed. This failure behaviour highlights the poor adhesion at the fibre–matrix interface when untreated recycled fibres are employed. In contrast, the failure region of the T-RC sample exhibits a distinct and well-defined failure region where individual fibres are clearly visible. This is due to the beneficial effects of the chemical treatment, which effectively dissolves the residues of old matrix from the fibre surface and within the fibre bundles. Therefore, in this failure region, minimal fibre pull-out occurs, and no fibre debonding is observed. This aspect, in conjunction with the results from the mechanical tests, highlights a significant improvement in fibre–matrix adhesion that can be associated with the enhanced surface characteristics of the treated fibres.

## 4. Conclusions

This study was focused on the combined effect of a mechanical and a soft solvothermal recycling method performed at autoclave-like temperatures on CFRP laminates from the aeronautic sector. The presence of flame-retardant agents within the matrix system increases the complexity of the chemical process in terms of matrix dissolution efficiency. In the first part of the study, the CFRP materials were machined with a milling process adopting a low spindle speed and high feed rate to maximise the MRR while minimising cutting power and energy consumption. Part of the machined material was subjected to the two-step low-temperature chemical treatment with both acetic acid and acetone-based solutions.

At the end of the experimental campaign, it is possible to point out that:

- The milling process can be considered a suitable method to recycle the end-of-life composite materials. The selected process parameters promoted the formation of fibre-rich chips like fine fibres, minimising resin-rich powder production. More than the 75% of the processed material was fine fibres, while powders accounted for only 16%.
- The two-step soft chemical treatment enhanced the fibre bundle’s swelling and matrix dissolution, achieving swelling ratio and mass loss values of approximately 55% and 24%, respectively.
- The FTIR analysis confirmed the total removal of the epoxy resin and the flame-retardant agents from the fibre’s surface testified by the absence of all the bands between 1014 cm^−1^ and 1600 cm^−1^. However, the presence of the 1014 cm^−1^ band indicates a partial fibre’s oxidation that in turn promotes improved fibre–matrix interaction and adhesion.
- The flexural tests revealed a significant increase in the mechanical properties of the T-RC sample because of the additional soft solvothermal recycling process in improving the flexural performance of the recycled composite material. Flexural strength and modulus increased by 42.24% and 76.25%, respectively, due to the enhanced adhesion from the additional chemical treatment.
- Microscopic inspection revealed strong bonding in the T-RC sample with no visible debonding and a minimal fibre pull-out, while the U-RC sample exhibited weak bonding with the instauration of these phenomena. These findings confirm that the soft solvothermal process improves fibre–matrix interaction, leading to superior mechanical performance and highlighting the importance of chemical surface modification in recycled composites.

## Figures and Tables

**Table 1:** Main characteristics of the produced samples.

Label	Reinforcement	Chemical Treatment	Fibre Weight Percentage [%]	Thickness [mm]
**U-RC**	Fine fibres	-	35.00	2.77
**T-RC**	Fine fibres	Two-step solvolysis	35.00	2.75

## Data Availability

The raw data supporting the conclusions of this article will be made available by the authors on request.
